# Genetic and Environmental Effects on the Early Motor Development as a Function of Parental Educational Attainment

**DOI:** 10.1249/MSS.0000000000003209

**Published:** 2023-05-12

**Authors:** YAHUA ZI, CATHARINA E. M. VAN BEIJSTERVELDT, MEIKE BARTELS, ECO J. C. DE GEUS

**Affiliations:** 1 School of Exercise and Health, Shanghai University of Sport, Shanghai, CHINA; 2 Department of Biological Psychology, Vrije Universiteit Amsterdam, Amsterdam, THE NETHERLANDS; 3 Netherlands Twin Register, Department of Biological Psychology, Vrije Universiteit Amsterdam, THE NETHERLANDS; 4 Amsterdam Public Health Research Institute, Amsterdam, THE NETHERLANDS

**Keywords:** MOTOR DEVELOPMENT, TWINS, HERITABILITY, GENETICS, SEX DIFFERENCES

## Abstract

**Introduction:**

The contribution of genetic and environmental factors to individual differences in early motor development is still largely uncharted. This large-scale twin study establishes the genetic and environmental influences on the timing of motor milestones achievement, and it further tests whether the influences are moderated by parental education.

**Methods:**

The twins came from families registered in the Netherlands Twin Register (NTR) from 1986 to 2016. In 30,256 complete twin pairs, mother-reported ages at which each twin was able to first-time roll from back to belly, sit unassisted, hands-and-knees crawl, stand up unaided, and walk independently were used to extract an early motor development factor. Parental education was dichotomized (“both parents with low/average education” vs “at least one parent with high education” with university degree as a threshold).

**Results:**

Additive genetics explained 52% of the variance in motor development, the remaining 39% and 9% were explained by shared and nonshared environment separately. Mean age of achieving motor milestones tended to be higher in infants with high educated parents, and a moderation of parental education on the genetic and environmental variance in motor development was seen in female twins with larger heritability in the high educated parents group (64% vs 43%) paired to a lower shared environmental influence (28% vs 48%). Only 7%–8% of the variance was accounted for nonshared environmental factors, including measurement error. The pattern of results did not change when the degree of urbanicity, a correlate of parental education, was additionally considered.

**Conclusions:**

Genetic factors explain most of the individual differences in the timing of motor milestone achievement, but factors related to the shared home environment also play an important role in early motor development.

Motor milestones, such as rolling over, sitting without support, crawling, standing unaided, and walking unsupported, provide a framework for observing and monitoring the development of a child and help recognize those who may be at risk for development delay (1). Early achievement of these developmental milestones predicts increased physical activity (PA) (2,3), more sports participation (4), and decreased sedentary behavior (5) in childhood and adolescence. Motor milestones have also been associated with nonmotor outcomes, including cognitive ability (6–8), educational level (9), and language acquisition (10) and with general health outcomes, such as weight status and physical fitness (11).

A better understanding of the etiology of motor milestones development is an important basis for designing interventions for infants lagging in their motor milestone achievement. Two classes of potential determinants are genetic and shared environmental factors. Genetic factors can be related to effects on central and peripheral motor control, including functional connectivity of sensory and motor areas, neural motor pathways, and the neuromuscular connections. Shared environment is mainly family environment for infants, which includes 1) physical opportunities and evocative challenges for motor activities provided by the home environment and 2) parental behaviors, like modeling, coactivity, and encouragement of motor activities (12,13). Conventional cohort studies, including studies that relate parental characteristics to offspring motor development, do not allow the disentanglement of resemblance into genetic (“nature”) and shared environmental (“nurture”) influences (14). Twin studies do allow such disentanglement, and if the shared environment does not play a large role, genetic effects could be further decomposed into additive and nonadditive genetic effects.

To date, only a few twin studies have examined the heritability of the timing of motor milestone achievement, and the large heterogeneity in the results makes it difficult to draw firm conclusions (for related twin studies, see Table 1). Peter and colleagues (15) reported variance decomposition of the month in which motor milestones were achieved among a sample of 186 twins (58 monozygotic [MZ] and 128 dizygotic [DZ] twins) and 480 siblings. They found that the shared environmental influences explained the majority of the variation in ages achieving sitting without support, turning over, and walking five steps unaided. By contrast, Goetghebuer et al. (16) showed in 44 MZ and 124 DZ twins that over 90% of the variance observed for crawling, sitting up, standing, and walking was due to genetic rather than environmental factors. Differences in findings may well have been due to the small sample sizes. In a much larger sample, Smith et al. (17) reported that the age of first sitting (1247 MZ and 2705 DZ twins) and crawling (1174 MZ and 2502 DZ twins) was equally influenced by the shared environmental influences (33%–42%) and genetic influences (48%–54%), whereas genetic influences (84%) dominated for the age of making the first steps (868 MZ and 1976 DZ twins).

**TABLE 1:** Heritability of motor milestones: an overview of twin studies, ranked by published year.

Reference	Traits	Age, Mean ± SD, months	Sample	Country	Instrument	Estimation Method	A (%)	C (%)	E (%)
Peter et al. (15)	Turn over	4.7 ± 1	58 MZ twins/128 DZ twins	Israel	Multiple survey items	Variance*^a^*	33.5	50.5	16.1
	Sit up	7.8 ± 1	58 MZ twins/128 DZ twins	Israel	Multiple survey items	Variance*^a^*	31.2	56.0	12.8
	Stand up	8.7 ± 2	58 MZ twins/128 DZ twins	Israel	Multiple survey items	Variance*^a^*	0	33.3	66.7
	Walk (5 steps)	13.3 ± 2	58 MZ twins/128 DZ twins	Israel	Multiple survey items	Variance*^a^*	22.2	66.6	11.2
Goetghebuer et al. (16)	Turn over	5.0	44 MZ twins/124 DZ twins	UK	Denver Development Screening Test (DDST)	Correlation	0	–*^b^*	–*^b^*
	Crawl	7.2	44 MZ twins/124 DZ twins	UK	DDST	Correlation	93	–*^b^*	–*^b^*
	Sit without support	7.4	44 MZ twins/124 DZ twins	UK	DDST	Correlation	94	–*^b^*	–*^b^*
	Stand with support	8.9	44 MZ twins/124 DZ twins	UK	DDST	Correlation	72	–*^b^*	–*^b^*
	Walk with support	9.8	44 MZ twins/124 DZ twins	UK	DDST	Correlation	90	–*^b^*	–*^b^*
Smith et al. (17)	Sit	7.3 ± 2	1247 MZ twins/2705 DZ twins	UK	Multiple survey items	SEM*^c^*	48	42	10
	Crawl	9.3 ± 2	1174 MZ twins/2502 DZ twins	UK	Multiple survey items	SEM*^c^*	54	33	13
	Walk	13.1 ± 2	868 MZ twins/1976 DZ twins	UK	Multiple survey items	SEM*^c^*	84	0	16

*^a^*Adjusted for gestational age.

*^b^*Not estimated.

*^c^*Adjusted for gestational age, age at questionnaire completion, and sex.

SEM, structural equation modeling; Variance, variance components analysis; A, variance explained by genetic effect; C, variance explained by shared environmental effect; E, variance explained by specific (or nonshared) environmental effect; Turn over, the months of the twin for the first time being able to roll over from back to belly; Sit, the months of the twin for the first time being able to sit without support; Crawl, the months of the twin for the first time being able to crawl on hands and knees; Stand, the months of the twin for the first time being able to stand; Walk, the months of the twin for the first time being able to walk.

Two potential sources for the shared environmental influences on motor milestones are the (physical) home environment and parental behavior (18–21). Both of these sources may vary as a function of parental educational attainment with lower educated parents possibly providing homes with lack of enough space, less infant equipment, and more child time in immobilized position, which were reported to affect infants motor development (21,22). An added complexity is that parental education may be correlated with urbanicity, itself a potential determinant of motor development (23,24). Areas with higher urbanization are exposed to higher population density, denser buildings, less accessibility of green space, and less perceived safety, resulting in inequalities in health (25,26) and potentially in motor development. In the present study, we used a very large data set on motor milestones in the Netherlands Twin Register (NTR) to assess genetic and environmental influences on an overall motor development factor (MD-FS) based on five important early motor milestones: the age of first-time sitting without support, crawling on hands and knees, rolling from back to belly, standing without support, and walking without support. To detect a potential role for parental educational attainment, we compared the genetic and shared environmental contribution to the child’s motor development in parents with low/average versus high educational attainment and formally test their moderation through gene-by-environment twin modeling (27). We repeated the analyses after taking urbanicity into account.

## METHODS

### Participants

The study included participants enrolled in the young NTR (YNTR), a longitudinal, large population-based, and ongoing cohort that has recruited newborn twins for more than three decades (28). NTR systematically approached parents to register their newborn twins in the YNTR, and between 30% and 40% of all Dutch twin pairs born from 1986 until 2016 were registered (CBS; https://www.cbs.nl/en-gb/figures/detail/37422eng?q=twins/). When parents were willing to participate, they received a survey in the months following registration about the course of pregnancy, the twins’ birth, and the early developmental characteristics. The survey included a one-page notebook with a list of the motor milestones that they were asked to keep track of. If the survey had been returned, the mother (and in more recent years also the father) would receive a second survey that included questions about motor development by the time twins reach their second birthday (29). The average response rate of the mothers across the three decades was 65%.

The survey was completed by mothers for 30,256 complete twin pairs, when the twins were 28.2 ± 3.6 months old (range, 14.4–36.0 months). To determine whether same-sex twins were MZ or DZ, a number of questions were asked about their physical resemblance (e.g., “Do the children resemble each other?” with answer options “yes, they are barely different,” “yes, but well distinguishable,” “no, not a lot,” and “no, not at all”) as detailed in full elsewhere (28). The accuracy of zygosity determination from these questions is 93.8% compared with DNA markers or blood typing (28). The distribution of boys/girls was 49.9%/50.1%. The distribution of the zygosity was 34.1% and 65.9% for MZ and DZ twins, respectively (of which 33.2% were opposite sex twins). For 398 pairs, the mother reported on the motor milestones when the child was more than 3 yr old—these pairs were discarded to avoid bias due to the longer recall interval.

Recruitment procedures and surveys sent to the parents of the twins were approved by the Medical Ethical Review Committee of the Vrije Universiteit Medical Center Amsterdam. Completion and return of the filled-out surveys by the parents were considered to signal active informed consent.

### Measures

#### Early motor milestones

With the first survey, parents had received a one-page notebook with a list of the motor milestones that they would be asked about in the second survey. This allowed parents to write down the timing in the notebook, as soon as the milestone was achieved. Data obtained by this prospective recording method were shown to be reliable (29). In the second survey, parents were asked to use the notebook and to report the age of obtaining motor milestones: “With how many months could your twin for the first time—roll over from back to belly (turn), sit without support (sit), crawl on hands and knees (crawl), stand without support (stand), and walk without support (walk)?” 88.8% of the mothers returned the motor milestones recording notebook; missing values for the various motor milestones were between 3.5% and 8.5%.

When only one single motor milestone was missing, we imputed that missing milestone by the population mean. This increased the sample available for a principal-components factor analysis that was used to summarize the correlational structure of the items in higher order factors. Using an eigenvalue of 1 as the cutoff, all five motor milestone items strongly loaded on a single factor. Varimax rotation was used to derive the orthogonal factor scores for this overall MD-FS. Only one factor exceed the eigenvalue criteria of 1, and this factor explained a cumulative variance of 61.3% of the variance in the five items.

#### Parental educational attainment

Parents of children in the YNTR were asked to report the highest education level that they had attained at the time their twins were born. Parental educational attainment was used to classify both mothers and fathers into two levels, “low or average education” (coded 1, 69.6% of the mothers and 66.1% of the fathers) and “high education” (coded 2, 30.4% of the mothers and 33.9% of the fathers). “High education” corresponds to a university degree or a university of applied sciences degree. The parental educational attainment data were combined into two categories: “both parents with low or average education” (54.9%) and “at least one parent with high education” (45.1%) (30).

#### Urbanicity

The family’s postal zone at the time of the second survey was used as a proxy for the postal zone where they lived during the first 2 yr of the infants. A small percentage (12%) of the families had moved to a different address after birth and before the second survey, but we had no records of the exact timing of the address change. Based on the postal zone of the second survey, the address density of their living area (OAD) was obtained, which is the average number of addresses per kilometer square (km^2^). The urbanicity level of the family’s living area was classified into two levels, urban and rural areas, according to 4-digit OAD. The present classification of urbanicity is adapted from the five-category method: very strongly urban, strongly urban, moderately urban, few urban, and nonurban (CBS; https://www.cbs.nl/nl-nl/maatwerk/2006/08/toelichting-kerncijfers-postcodegebieden-2004). “Urban area” (coded 2) indicates OAD larger than or equal to 1000 and “rural area” (coded 1) less than 1000. Reliable postal zone information was not available for all years of data collection, and not all parents provided active permission for record linkage to postal zone information. Hence, the data set with urbanicity information was only 34.3% of the total data set. In the data set with urbanicity information, 40.2% of the families were living in an urban area and 59.8% in a rural area.

#### Covariates

Analyses of motor milestones were adjusted for sex, birth order of the twins, gestational age, and parental education attainment. The information of sex, birth order, and gestational age was obtained by maternal reports from the first survey in the subsequent months after twins born. As a sensitivity analysis, we further repeated the analyses also correcting for urbanicity in the subset of families with relevant data.

### Statistical Analyses

The main effects of the covariates were tested by regressing them on the MD-FS score using family as the cluster variable to account for the nested structure of the data (R package *gee*). For all the analyses and model-fitting procedures, the threshold for significance was set at *α* = 0.01. Twin correlations were estimated on the MD-FS after the covariate effects had been regressed out using the UMX residualised function of the R package *umx*.

The decomposition of the variance into genetic and environmental sources in twin studies is based on the comparison of similarity of MZ and DZ twin pairs. MZ twins originate from the same fertilized egg, meaning that they are nearly genetically identical, whereas DZ twins share on average 50% of their segregating additive genetic variation. When the similarity of MZ twins (quantified by the MZ correlations) is around two times larger (or more) than that of DZ twins, this constitutes evidence for genetic influences on the phenotype of interest. However, when the similarity of MZ and DZ is more alike, this constitutes evidence for shared environmental influences. The relative importance of the “latent” genetic and environmental influences can be derived by structural equation modeling from observed covariances in both twin types.

Genetic structural equation modeling was done in OpenMx 2.20.7 under R 4.2.2 (R Development Core Team, 2022). First, it was tested whether parsimonious models that equated means and variances of MD-FS among MZ and DZ twins sufficiently fitted the data to simplify further genetic modeling. Next the total phenotypic variance of motor milestones was divided into sources of additive genetic variance (A), dominant (or called nonadditive) genetic variance (D), shared (or common) environmental variance (C), or nonshared (or person-specific or unique) environmental variance (E), while correcting for sex, birth order of the twin, gestational age, and parental education effects on the mean. E includes measurement error. Because C and D effects cannot be estimated simultaneously in the classical twin model, we selected models including C (i.e., ACE model) as this was the hypothesis of interest. This choice was also in keeping with the ratio of the MZ correlation to DZ correlation, which was lower than 0.5. In general, only when the MZ correlation is much larger than twice of DZ correlation (i.e., rMZ > 2 × rDZ), an ADE model is more appropriate than an ACE model. A number of nested models were fitted to the data, which tested for qualitative and quantitative sex differences as described before by Huppertz et al. (30). Estimates for the variances of A, C, and E effects on the trait (here for the overall MD-FS) from the best-fitting model were then extracted, separately for males and females when appropriate.

As a sensitivity analysis, we repeated the above model-fitting steps using urbanicity as an additional covariate. Note that this reduced the sample size to only one-third of the data used in the above models.

**FIGURE 1:**
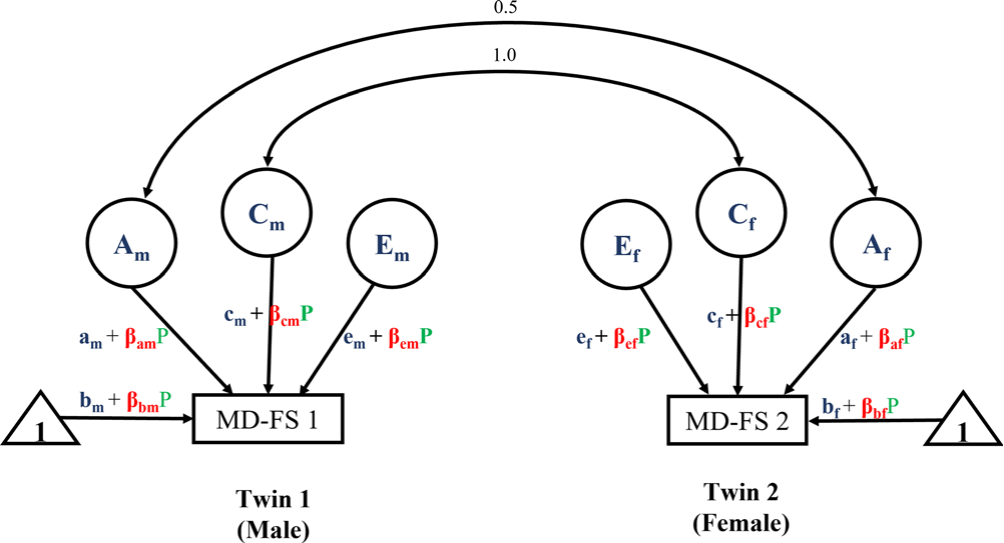
The moderated univariate ACE pathway model with quantitative sex difference. Where MD-FS 1 and MD-FS 2 represent the MD-FS score of twin 1 and twin 2; A_m_, C_m_, E_m_, A_f_, C_f_, and E_f_ are additive (A), common environmental (C), and nonshared environmental (E) components decomposed from trait (here for MD-FS) variance for males and females, respectively; a_m_, c_m_, e_m_, a_f_, c_f_, and e_f_ represent male- or female-specific path coefficients of A, C, and E components; b_m_ and b_f_ are the path coefficients for the mean of the trait among males and females; *β*_am_, *β*_cm_, *β*_em_, *β*_bm_, *β*_af_, *β*_cf_, *β*_ef_, and *β*_bf_ stand for the corresponding moderation parameters of A, C, E, and mean among males and females, respectively; P is the moderation parameter (here for parental education level); the covariance of additive genetic (A) and common environmental (C) effects between twin 1 (male) and twin 2 (female) are 0.5 and 1.0 separately.

To examine moderation of the genetic and environmental influences on the variance by parental educational attainment level, the above analysis strategy was repeated twice, first using the twins stemming from a family with low/average parental education and second using the twins stemming from a family with high parental education. When estimates for A, C, or E in the twins with low/average parental education fell outside of the confidence intervals for estimates for twins with high parental education, or *vice versa*, this points to a moderation effect of parental educational level on the A, C, or E variance components. Formal testing of this moderation then ensued, using the univariate moderation model depicted in Figure 1, to test the moderation of by parental education of the paths from the latent factors A, C, and E to the motor milestones. Because the genetic correlation between moderator and motor milestones was very low (R*g* = 0.06), a univariate model was deemed appropriate as it yields more power than a bivariate model that explicitly models the gene-by-moderator correlation (27,31).

In Figure 1, *β*_bm_ and *β*_bf_ stand for the effect of parental education on the mean of MD-FS. After the variance common to MD-FS and parental education is controlled, the variance of MD-FS is decomposed into estimates of A, C, and E, each of which is expressed as a linear function of parental education. The A pathway is estimated by the equation, a_m_ + *β*_am_ × P and a_f_ + *β*_af_ × P for males and females, with a_m_ and a_f_ representing the constant additive genetic influences and *β*_am_ and *β*_af_ denoting the moderating effect of parental education on the additive genetic influences. Analogous treatment applies to the C and E components. The significance of moderation was tested by consecutively constraining the moderation of A, C, and E to zero and evaluating the reduction of model fit in relation to the full model which allowed moderation on A, C, and/or E components.

As a sensitivity analysis in the subset of the data with urbanicity data, we repeated the moderation model using urban versus rural as the moderator variable.

## RESULTS

Table 2 depicts the available complete twin pairs and the mean and SD values of the age (in months) at which motor milestones were achieved as reported by the mother for each zygosity group, stratified by parental education level. The table also presents the MD-FS score based on the five milestones jointly (MD-FS). As expected, the mean values of the first-time achievement of the motor milestones tended to increase from rolling from back to belly, sitting without support, crawling on hands and knees, standing without support, to walking independently in each group.

**TABLE 2:** Mean and SD values of the age of achieving the motor milestones (in months) reported by the mother, stratified by zygosity and parental education attainment level.

Motor Milestones	Total		Low/Average Parental Education		High Parental Education		*P*
	Mean Age ± SD	*n*	Mean Age ± SD	*n*	Mean Age ± SD	*n*	
All		30,256		12,227		10,063	
MZM							
First rolling from back to belly (months, IQR = 5.0–7.0)	6.22 ± 1.68	3,802	6.24 ± 1.71	1573	6.29 ± 1.59	1376	0.21
First sitting without support (months, IQR = 7.5–10.0)	8.89 ± 1.93	3,811	8.92 ± 1.94	1584	9.02 ± 1.82	1375	0.04
First crawling on hands and knees (months, IQR = 8.5–11.5)	10.19 ± 2.32	3,806	10.16 ± 2.32	1572	10.44 ± 2.22	1375	2.6e−06*
First standing without support (months, IQR = 11.0–14.0)	12.63 ± 2.68	3,713	12.42 ± 2.63	1544	12.95 ± 2.67	1338	5.0e−14*
First walking without support (months, IQR = 13.5–17.0)	15.29 ± 2.45	3,868	15.21 ± 2.47	1585	15.47 ± 2.34	1398	2.7e−05*
Motor Development Factor Score (IQR = −0.59 to 0.71)	0.10 ± 1.01	3,614	0.08 ± 1.00	1501	0.22 ± 0.97	1308	1.2e−07*
DZM							
First rolling from back to belly (months, IQR = 5.0–7.0)	6.16 ± 1.71	3,822	6.22 ± 1.69	1708	6.16 ± 1.64	1267	0.16
First sitting without support (months, IQR = 7.5–10)	8.72 ± 1.89	3,830	8.78 ± 1.90	1705	8.74 ± 1.82	1273	0.46
First crawling on hands and knees (months, IQR = 8.5–11.0)	10.02 ± 2.34	3,809	10.01 ± 2.36	1698	10.20 ± 2.29	1268	0.001*
First standing without support (months, IQR = 10.5–14.0)	12.41 ± 2.71	3,764	12.28 ± 2.68	1676	12.61 ± 2.69	1252	2.2e−06*
First walking without support (months, IQR = 13.0–16.5)	14.99 ± 2.44	3,919	14.90 ± 2.44	1726	15.12 ± 2.37	1316	0.0004*
Motor Development Factor Score (IQR = −0.68 to 0.57)	0.01 ± 1.01	3,626	−0.01 ± 1.01	1633	0.07 ± 0.99	1206	0.006*
MZF							
First rolling from back to belly (months, IQR = 5.0–7.0)	6.22 (1.68)	4077	6.32 (1.74)	1776	6.22 (1.52)	1440	0.008*
First sitting without support (months, IQR = 7.5–10.0)	8.73 (1.84)	4093	8.83 (1.87)	1787	8.81 (1.69)	1446	0.72
First crawling on hands and knees (months, IQR = 8.5–11.0)	10.13 (2.29)	4077	10.15 (2.30)	1774	10.30 (2.21)	1443	0.009*
First standing without support (months, IQR = 11.0–14.0)	12.62 (2.71)	4018	12.48 (2.71)	1759	12.96 (2.68)	1409	2.8e−12*
First walking without support (months, IQR = 13.5–16.5)	15.13 (2.44)	4175	15.05 (2.40)	1810	15.30 (2.41)	1475	1.7e−05*
Motor Development Factor Score (IQR = −0.61 to 0.66)	0.06 (1.01)	3878	0.07 (1.01)	1699	0.15 (0.96)	1364	0.0005*
DZF							
First rolling from back to belly (months, IQR = 5.0–7.0)	6.19 (1.74)	3533	6.25 (1.75)	1610	6.16 (1.69)	1127	0.05
First sitting without support (months, IQR = 7.5–9.0)	8.53 (1.81)	3529	8.55 (1.83)	1610	8.66 (1.70)	1127	0.02
First crawling on hands and knees (months, IQR = 8.5–11)	9.95 (2.29)	3516	9.88 (2.26)	1599	10.21 (2.23)	1124	8.3e−08*
First standing without support (months, IQR = 10.5–14.0)	12.29 (2.65)	3455	12.07 (2.59)	1577	12.69 (2.63)	1104	<2.2e−16*
First walking without support (months, IQR = 13.0–16.0)	14.86 (2.43)	3606	14.73 (2.41)	1633	15.10 (2.38)	1162	1.0e−08*
Motor Development Factor Score (IQR = −0.75 to 0.51)	−0.06 (1.00)	3341	−0.09 (0.99)	1526	0.06 (0.97)	1069	2.1e−08*
DOS							
First rolling from back to belly (months, IQR = 5.0–7.0)	6.15 (1.70)	7456	6.19 (1.71)	3263	6.16 (1.65)	2453	0.23
First sitting without support (months, IQR = 4.5–9.0)	8.59 (1.84)	7497	8.64 (1.84)	3269	8.68 (1.74)	2467	0.21
First crawling on hands and knees (months, IQR = 5.0–10.5)	9.94 (2.28)	7429	9.93 (2.25)	3243	10.14 (2.28)	2448	7.6e−07*
First standing without support (months, IQR = 10.5–13.0)	12.25 (2.63)	7313	12.12 (2.60)	3202	12.56 (2.62)	2406	<2.2e−16*
First walking without support (months, IQR = 11.0–16.0)	14.87 (2.40)	7638	14.79 (2.38)	3290	15.06 (2.36)	2533	1.1e−09*
Motor Development Factor Score (IQR = −0.72 to 0.51)	−0.06 (0.98)	7059	−0.07 (0.97)	3099	0.03 (0.96)	2331	3.4e−08*

*P* values in the last column are for the difference between low/average and high parental education. *P < 0.01.

*N*, number of complete twin pairs, representing the pairs number of twins without missing data in both twins; MZM, monozygotic male twins; DZM, dizygotic male twins; MZF, monozygotic female twins; DZF, dizygotic female twins; DOS, dizygotic opposite twins; IQR, interquartile range.

When examining the effect of parental education (see *P* values in last column), an unexpected finding emerged such that a slower motor development was seen in infants from high educated parents. Twins in high parental education families achieved the final three motor milestones (crawl, stand, and walk) and overall MD-FS significantly later than that in low/average parental education families in nearly all zygosity-by-milestone combinations. The parental educational attainment effect was not present for first rolling from back to belly and for sitting without support.

For the covariates, the birth order of twins (*β*_birthorder–FS_ = 0.02, *P* = 0.062) did not significantly influence motor development, but sex (*β*_sex–FS_ = −0.04, *P* = 0.00023), child’s age at time of mother reporting (*β*_age–FS_ = −0.065, *P* = 0.001), gestational age (*β*_gestational age–FS_ = −0.12, *P* < 10^−30^), and parental education (*β*_PE–FS_ = 0.11, *P* < 10^−26^) did. Girls reached the motor milestones earlier than boys. Lower MD-FS were reported when the child was older at the time of mother reporting, which may reflect a recall bias such that the longer the time that elapsed since the actual motor milestone event, the more mothers tend to remember the milestone as having occurred earlier. A longer gestation was associated with faster motor development. Gestational age did not explain the parental education effect as it was not different between low and high educated parents groups, except in the twins of opposite sex, where it was 36.6 wk in the low/average parental education group and 36.8 wk in the high parental education group (*P* < 0.0001). In the subset of the infants for which postal zone data were available, living in urban areas was associated with faster motor development than living in rural areas (*β*_urban–FS_ = −0.068, *P* = 0.00043). Urbanicity was positively correlated with parental education level (polychoric *r*_urban–PE_ = 0.14, *P* = 0.006), indicating that the higher educated parents more often lived in urban areas. In keeping, adding an urbanicity by parental education interaction term yielded a significant interaction effect (*β*_urban*PE–FS_ = 0.12, *P* = 0.00023), showing that the fastest motor development occurred in infants from low to average educated parents living in urban areas.

### Twin correlations for motor milestones

**FIGURE 2:**
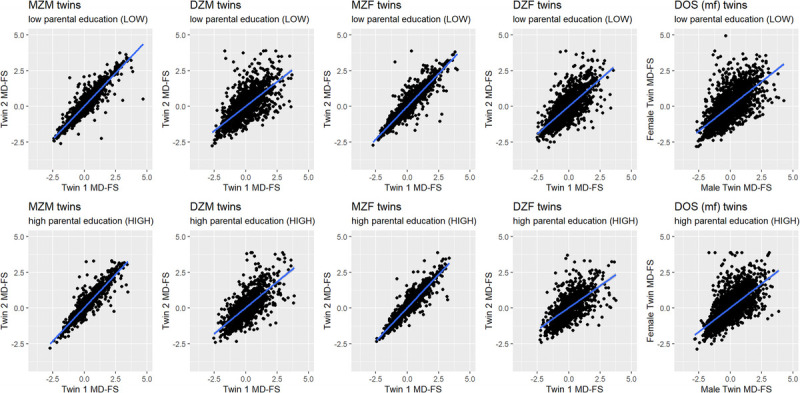
The full twin–twin plots for the overall MD-FS in each group, stratified by zygosity and parental education.

Table 3 depicts the raw and covariate-corrected twin correlations of the five motor milestone items and MD-FS, separated for low and high educational attainment of the parents. The first set of corrected twin correlations takes into account the effects of sex, gestational age, parental education, and birth order of the twins, and the second set adds urbanicity as an additional covariate. Figure 2 gives the full twin–twin plots for the general MD-FS score in each group.

**TABLE 3:** The observed and covariate-corrected twin correlations of five motor milestone items and the overall motor milestones factor score (MD-FS), separated for low/average and high educational attainment of the parents.

Parental Education	Turn		Sit		Crawl		Stand		Walk		MD-FS	
	*r* **_obs_**	*r***_cor_**/*r***_cor+_**	*r* **_obs_**	*r***_cor_**/*r***_cor+_**	*r* **_obs_**	*r***_cor_**/*r***_cor+_**	*r* **_obs_**	*r***_cor_**/*r***_cor+_**	*r* **_obs_**	*r***_cor_**/*r***_cor+_**	*r* **_obs_**	*r***_cor_**/*r***_cor+_**
MZM												
All	0.873	0.874/0.894	0.922	0.920/0.937	0.872	0.871/0.884	0.919	0.921/0.927	0.902	0.905/0.915	0.915	0.905/0.923
Low/average	0.866	0.868/0.903	0.907	0.907/0.933	0.859	0.859/0.862	0.914	0.913/0.920	0.899	0.897/0.914	0.905	0.894/0.918
High	0.883	0.882/0.888	0.936	0.936/0.940	0.883	0.886/0.900	0.928	0.928/0.931	0.913	0.913/0.915	0.927	0.919/0.927
DZM												
All	0.676	0.662/0.664	0.808	0.806/0.808	0.645	0.626/0.617	0.736	0.731/0.715	0.638	0.634/0.585	0.705	0.662/0.634
Low/average	0.664	0.661/0.693	0.803	0.799/0.806	0.634	0.631/0.624	0.713	0.712/0.709	0.638	0.636/0.586	0.685	0.660/0.638
High	0.669	0.666/0.634	0.818	0.817/0.810	0.616	0.617/0.608	0.750	0.752/0.722	0.629	0.630/0.583	0.711	0.665/0.629
MZF												
All	0.882	0.877/0.854	0.922	0.930/0.925	0.872	0.872/0.888	0.922	0.933/0.932	0.899	0.909/0.916	0.918	0.916/0.916
Low/average	0.882	0.878/0.858	0.932	0.932/0.928	0.880	0.876/0.897	0.923	0.924/0.928	0.901	0.904/0.915	0.921	0.911/0.914
High	0.876	0.875/0.849	0.927	0.926/0.922	0.871	0.867/0.880	0.944	0.943/0.934	0.917	0.915/0.916	0.933	0.924/0.918
DZF												
All	0.687	0.673/0.665	0.828	0.826/0.835	0.644	0.640/0.665	0.724	0.722/0.738	0.641	0.634/0.646	0.698	0.658/0.672
Low/average	0.696	0.705/0.689	0.831	0.834/0.856	0.672	0.672/0.696	0.735	0.743/0.750	0.660	0.661/0.675	0.723	0.694/0.702
High	0.631	0.625/0.646	0.814	0.813/0.813	0.587	0.592/0.635	0.683	0.685/0.721	0.595	0.591/0.617	0.647	0.607/0.647
DOS												
All	0.670	0.662/0.656	0.780	0.771/0.784	0.615	0.605/0.614	0.691	0.687/0.682	0.599	0.603/0.599	0.666	0.631/0.645
Low/average	0.669	0.669/0.681	0.776	0.776/0.788	0.622	0.621/0.610	0.698	0.698/0.695	0.604	0.604/0.615	0.675	0.643/0.649
High	0.653	0.654/0.633	0.764	0.764/0.780	0.586	0.584/0.615	0.670	0.667/0.667	0.596	0.598/0.582	0.652	0.615/0.641

*r*_obs_, the observed twin correlation; *r*_cor_, the twin correlation corrected with covariates: sex, gestational age, birthorder, and parental education attainment; *r*_cor+_, the twin correlation corrected with covariates: sex, gestational age, birthorder, parental education attainment, and urbanicity.

Turn, the months of the twin for the first time being able to roll over from back to belly; Sit, the months of the twin for the first time being able to sit without support; Crawl, the months of the twin for the first time being able to crawl on hands and knees; Stand, the months of the twin for the first time being able to stand without support; Walk, the months of the twin for the first time being able to walk without support; MZM, monozygotic male twins; DZM, dizygotic male twins; MZF, monozygotic female twins; DZF, dizygotic female twins; DOS, dizygotic opposite twins.

The MZ correlations for the MD-FS were consistently higher than DZ correlations, and MZ correlations were smaller than the twice of the same-sex DZ correlation, implying that both genetic and shared environmental effects contribute to the variance of motor milestone achievement. DOS correlations were only slightly lower than the same-sex DZ correlation, suggesting very small qualitative sex differences at best. As shown in the columns labeled *R*_cor/cor+_ in Table 3, correction for the covariates, including urbanicity, did not noticeably change the correlations in the five zygosity groups for the full group of twins, or for the twins from low/average and high parental education families separately.

### The relative contribution of the ACE variance components to motor development

Table 4 contains the model-fitting indices of the full ACE model with quantitative and qualitative sex differences and compares it with the fit of nested and more parsimonious models. As no evidence for qualitative sex differences was found (rA found close to 0.5 and rC close to 1), the ACE model without qualitative sex differences was used as the new baseline model to test for quantitative sex differences in A, C, E, or combinations. For each model, the estimated contribution of the additive genetic, shared, and nonshared environmental components to the variance in the MD-FS are given. The most parsimonious model that still fit the data well is an ACE model with quantitative sex differences because of small but significant differences between males and females mainly driven by a difference in nonshared environmental influences. Attempts to remove either A or C resulted in a very strong deterioration of the model fit, confirming that shared environmental and genetic factors both play a clear role in motor development.

**TABLE 4:** Model fit indices and genetic and environmental influences on the overall MD-FS for all infants and infants from low and high parental education families.

Parental Education	Baseline Model	Model	Model Fit Indices				Estimated Variance Components (%) [95%CI]					
			−2LL	*χ* **^2^**	∆*df*	*P*	A**_f_**	C**_f_**	E**_f_**	A**_m_**	C**_m_**	E**_m_**
All		Full ACE model	73,797.2	—	—	—	50.7 [50.3–51.1]	41.0 [40.6–41.3]	8.3 [8.3–8.3]	50.9 [50.5–51.3]	39.5 [39.1–39.9]	9.6 [9.6–9.7]
	Full ACE model	ACE model, no qualitative sex differences	73,800.2	3.066	1	0.08	52.3 [50.3–56.0]	39.4 [37.5–43.1]	8.3 [7.9–8.8]	52.5 [48.9–54.6]	37.9 [36.0–41.4]	9.6 [9.1–10.2]
	ACE model, no qualitative sex differences	ACE model, no quantitative sex differences for A, C, and E	73,814.4	14.145	3	0.003*	52.5 [51.6–53.5]	38.5 [37.6–39.4]	8.9 [8.6–9.2]	52.5 [51.6–53.5]	38.5 [37.6–39.4]	8.9 [8.6–9.2]
	ACE model, no qualitative sex differences	**ACE model with quantitative sex differences for E**	**73,803.7**	**3.438**	**2**	**0.179**	**52.8 [52.5**–**53.0]**	**38.8 [38.5**–**38.9]**	**8.5 [8.4**–**8.5]**	**52.2 [52.0**–**52.5]**	**38.4 [38.2**–**38.6]**	**9.4 [9.4**–**9.5]**
												
Low/average		Full ACE model	42,481.7	—	—	—	42.0 [41.5–42.5]	49.0 [48.5–49.5]	9.0 [9.0–9.0]	49.9 [49.3–50.4]	39.5 [38.9–40.0]	10.7 [10.6–10.7]
	Full ACE model	**ACE model, no qualitative sex differences**	**42,482.6**	**0.901**	**1**	**0.342**	**42.6 [41.1**–**44.2]**	**48.4 [43.8**–**50.0]**	**9.0 [8.6**–**9.2]**	**51.5 [49.9**–**52.9]**	**37.9 [36.6**–**39.5]**	**10.7 [10.3**–**11.1]**
	ACE model, no qualitative sex differences	ACE model, no quantitative sex differences for A, C, and E	42,503.7	21.09	3	0.0001*	47.7 [47.3–48.1]	42.5 [42.2–42.9]	9.8 [9.7–9.8]	47.7 [47.3–48.1]	42.5 [42.2–42.9]	9.8 [9.7–9.8]
												
High		Full ACE model	31,215.8	—	—	—	63.2 [61.9–64.5]	29.4 [28.2–30.6]	7.4 [7.1–7.6]	51.9 [50.6–53.1]	39.7 [38.5–40.9]	8.4 [8.2–8.7]
	Full ACE model	ACE model, no qualitative sex differences	31,216.3	0.438	1	0.508	64.9 [64.4–65.3]	27.8 [27.3–28.1]	7.4 [7.4–7.4]	52.3 [51.7–52.8]	39.3 [38.8–39.8]	8.4 [8.4–8.4]
	ACE model, no qualitative sex differences	ACE model, no quantitative sex differences	31,229.3	13.09	3	0.004*	59.1 [58.9–59.4]	33.0 [32.7–33.1]	7.9 [7.9–7.9]	59.1 [58.9–59.4]	33.0 [32.7–33.1]	7.9 [7.9–7.9]
	ACE model, no qualitative sex differences	**ACE with quantitative sex differences for A and C**	**31,219.9**	**3.631**	**1**	**0.057**	**64.2 [63.8**–**64.6]**	**28.0 [27.6**–**28.4]**	**7.7 [7.7**–**7.7]**	**53.2 [52.8**–**53.6]**	**38.8 [38.3**–**39.2]**	**8.0 [8.0**–**8.0]**

*P < 0.01.

Best fitting models are printed in bold.

**FIGURE 3:**
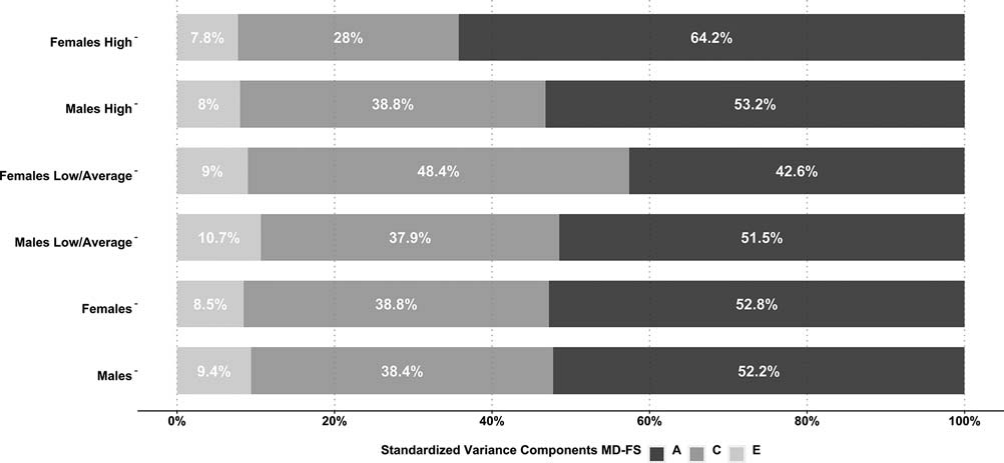
Standardized additive genetic, shared environmental, and nonshared environmental variance components for all infants, and split by parental education.

Figure 3 plots the standardized estimated variance components from this best-fitting ACE model. The standardized estimate for the contribution of additive genetic factors to the variances in MD-FS was 52.2% (95% CI = 52.0%–52.5%) and 52.8% (95% CI = 52.5%–53.0%) in males and females, respectively. The standardized estimate for the contribution of shared environmental contribution was 38.4% in males (95% CI = 38.2%–38.6%) and 38.8% (95% CI = 38.5%–38.9%) in females, leaving 9.4% of the male (95% CI = 9.4%–9.5%) and 8.5% of the female (95% CI = 8.4%–8.5%) variance in the timing of motor milestones achievement explained by nonshared environmental factors.

Adding urbanicity as an additional covariate did not change this pattern of findings. Results of this sensitivity analysis can be found in Supplemental Table 1 and Supplemental Figures 1 and 2 that plot the unstandardized and standardized estimated variances, with and without urbanicity as an additional covariate (see Supplemental Digital Content, http://links.lww.com/MSS/C863).

### Moderation of the ACE variance components by parental educational attainment

Next, we tested whether the variance components influencing motor development were moderated by parental education. As seen in Table 4, these models had to incorporate quantitative sex differences, as leaving them out would deteriorate model fit. In the low/average parental education group, standardized estimates for additive genetic effects on the MD-FS were 42.6% (95% CI = 41.1%–44.2%) in females and 51.5% (95% CI = 49.9%–52.9%) in males. Shared environmental effects explained 48.4% (95% CI = 43.8%–50.0%) of the variance in MD-FS in females and 37.9% (95% CI = 36.6%–39.5%) of male MD-FS variance. Estimates for the contribution of nonshared environmental factors were 9.0% (95% CI = 8.6%–9.2%) and 10.7% (95% CI = 10.3%–11.1%) in females and males, respectively.

Results in the high parental education yielded standardized estimates for A of 64.2% (95% CI = 63.8%–64.6%) in females and 53.2% (95% CI = 52.8%–53.6%) in males. For C, the female and male estimates were 28.0% (95% CI = 27.6%–28.4%) and 38.8% (95% CI = 38.3%–39.2%), and for E, 7.7% (95% CI = 7.7%–7.7%) and 8.0% (95% CI = 8.0%–8.0%).

Formal testing of the moderating role of parental education on the MD-FS with a univariate moderation model allowing quantitative sex differences confirmed the significant moderation of A, C, and E by parental education attainment, while yielding very similar estimates (see Table 5). Finally, sensitivity analysis adding urbanicity as an additional covariate did not change the findings on moderation by parental education.

**TABLE 5:** Moderation of A, C, and E effects on the overall MD-FS by parental education, split by sex.

Baseline Model	Model	Model Fit Indices			
		−2LL	*χ* **^2^**	∆*df*	*P*
Males					
	Full moderation model (ACE model with full moderation of A, C, and E)	73,705.55	—	—	—
Full moderation model	No moderation model (ACE model without moderation of A, C, and E)	73,753.89	48.33924	3	1.80e−10*
Full moderation model	No moderation of A model (ACE model with moderation of C and E, but not A)	73,706.46	0.9093372	1	0.34
No moderation of A model	**No moderation of A and C model (ACE model with moderation of E only)**	**73,709.76**	**3.296018**	**1**	**0.07**
No moderation of A and C model	No moderation model (ACE model without moderation of A, C, and E)	73,753.89	44.13388	1	3.07e−11*
Females					
	**Full moderation model (ACE model with full moderation of A, C, and E)**	**73,705.55**	—	—	—
Full moderation model	No moderation model (ACE model without any moderation)	73,757.75	52.20162	3	2.71e−11*
Full moderation model	No moderation of A model (ACE model with moderation of C and E, but not A)	73,734.63	29.07939	1	6.95e−08*
Full moderation model	No moderation of C model (ACE model with moderation of A and E, but not C)	73,737.95	32.39756	1	1.26e−08*
Full moderation model	No moderation of E model (ACE model with moderation of A and C, but not E)	73,731.43	25.88238	1	3.63e−07*

Model fit indices (−2LL, *χ*^2^, ∆df) and significance for the full moderation model and nested models constraining the unstandardized additive genetic (A), shared environmental (C), and nonshared environmental (E) variance component to be equal for infants of low and high educated parents.

*P < 0.01.

Best fitting models are printed in bold.

## DISCUSSION

In this study, we establish the relative contribution of genetic and environmental factors to individual differences in the achievement of five major motor milestones in the early childhood. We find that additive genetic factors explain the largest part of variation in these indices of overall motor development with a heritability estimate of 52% for the factor score capturing the timing of all five motor milestones. At the same time, we find a strong effect of shared environmental factors on milestone achievement, explaining 39% of the variance. When parental educational attainment level is taken into account, significant sex differences emerge in these estimates. The educational level of the parents moderates the heritability of motor milestone achievement in a sex-specific way such that genetic effects on the motor development of girls raised by higher educated parents (heritability = 64.2%) is stronger than that on girls raised by lower educated parents (heritability = 42.6%). In reverse, shared environmental effects are weaker in girls raised by higher educated parents than in girls raised by lower educated parents (28.0% vs 48.4%).

Findings are in line with the handful of earlier twin studies that examined the heritability of the timing of motor milestone achievement (15–17) and nominated both shared environmental and genetic influences as the major sources of variation in motor development. However, the 10-to-1000 times larger sample size of our study now resolves the large heterogeneity in the estimates from these previous studies. In our study, we find the largest contribution to motor development comes from additive genetic factors. These factors could be operating at multiple levels and include genetic variants with effects on early body composition (32), skeletal muscle development (33), peripheral nerve conduction speed (34), or neurodevelopmental delays in (sub)cortical sensorimotor structures and their connectivity (35).

Despite the importance of genetics, the shared home and family environment also weigh strongly on the timing of motor milestone development, explaining an additional 38.5% of the differences between the infants studied. The substantial influence of the child’s home and family environment may explain why motor milestone achievement appears to have been slowing down over time in the past decades (36–38). Our twin model can only establish that shared environmental factors are important, leaving the exact home and family environmental aspects that cause differences in motor development still to be established. Past research nominated increases in average parental educational attainment, urbanization, and technology expansion as possible candidates (23,39).

Various studies (18,40–42) showed that a lower level of parental education was associated with later motor development. This impaired motor development effect may be caused through the effects of the linked trait of low socioeconomic status (SES) on the affordances offered to 3- to 18-month infants, such as outside physical space and gross motor toys (20). The socioeconomic effects on motor development remain active over time. Ferreira et al. (41) showed that the motor development of school-age children (6 to 10 yr old) increases with SES, in which the main caregiver’s education level was included. Based on these past findings, we had the clear expectation that infants from high parental education families would develop motor skills earlier than that from low or average parental education families. In contrast to this expectation, later not earlier motor development was found in twins with high educated parents across all the zygosity groups. This was true for the factors score and three individual motor milestones, with first rolling from back to belly and first independently sitting being the exceptions.

We first considered a correlation between parental education and urbanicity as a possible explanation for these unexpected findings because higher levels of green space and composite nature can be helpful to locomotor development (23). However, the urbanicity of the home environment did not explain the parental education effect and may even have attenuated it. We found that living in urban areas was associated with faster motor development than living in rural areas, and that high educated parents in the Netherlands tend to live in the more urban areas. It remains unclear, therefore, why the infants of low-to-average educated parents in this large Dutch twin sample more rapidly gain motor milestones, such as first rolling, crawling, independently standing, and walking, but several possible mechanisms can be proposed. We note that the parental education effect is not likely to be mediated by pre- or perinatal complications, preterm births, and lower birth weight. A study on 3- to 12-month Canadian infants showed no association between maternal education and very early infant motor development (42). Our own data further argue against pre- or perinatal complications as a source of the parental education effect. Although we replicate the well-known effect that shorter gestation time leads to slower motor development (43), gestational age did not explain the parental education effect as it was not different between low and high educated parents groups. Furthermore, the birth order of twins, which is linked to lower birth weight in the second born (44), also did not significantly influence motor development. We used a fairly crude measure of urbanicity, a dichotomy based on the address density of the surrounding area, which will not capture the full spectrum of urbanicity, and be only imperfectly correlated to, e.g., familial SES (e.g., parental education), mixed-use land, or other characteristics of the neighborhood. Additional home environment information, such as the number of adults and children in the family, nutrition, illness situation/history, and accessibility to healthcare, may help explain the unexpected finding, but we had no solid record of this information.

One possible explanation for the unexpected parental education effect on the early motor development is that the high educated parents tend to observe the guidelines for sleeping position more than low educated parents, i.e., they place their infants in supine sleeping position to avoid sudden unexpected death (45,46). Another potential reason is that the high educated Dutch parents are apt to believe that motor development should be allowed to occur naturally rather than using active stimulating practices (e.g., placing infants in prone place when awake, the use of activity mat, baby bouncer, etc., equipment) (22,47). These practices and beliefs have been reported to predict lower infants’ motor development (21). Low or average educated parents might give more degree of freedom for motor exploration, thus helping toddlers attain self-locomotion skills (such as crawling, standing, and walking) (48). This, in turn, fundamentally changes the infants’ affordances, providing them novel possibilities for perception, exploration, and learning and instigating cascades of development in far-flung domains (49) that result in a reciprocal relation between motor development and affordances. It has also been reported that, compared with high educated parents, low educated parents in the Netherlands are better at creating a home environment that encourages free motor exploration, such as adequate stair gate and window sill guards (50), which is important for novice crawlers and walkers.

Given its effects on mean motor development, we further tested the idea that parental education attainment can act as a modifier of genetic and environmental effects on early motor development in infants. The genetic and environmental variances on the motor milestones factor scores were computed separately for infants with two low educated parents and infants with at least one high educated parent. Heritability of motor development was similar in male infants raised by low-to-average or by high educated parents. Female infants with high educated parents displayed a much stronger genetic influence on motor milestone attainment than female infants of low-to-average educated parents. The higher heritability was paired to a lower contribution of the shared home and family environment to motor development in infants with high educated parents. The female-specific moderation was further confirmed in a genetic moderation model that directly tested the moderation on the genetic and environmental path loadings (27). Our data provide no solid clues to an explanation for the higher heritability in females of high educated parents, as the unstandardized estimates show an absolute increase in genetic and decrease in shared environmental variance. They may point to a more uniform biological maturation process in children from high educated parents, possibly linked to genetic effects on cognitive ability, and they may be offered a more uniform shared home environment. Alternatively, a difference between low/average and high educated parents in the reporting bias might also contribute to parental education’s role as a modifier on the genetic and environmental effects on early motor development in infants.

Our findings have implications for research on the determinants of PA. Various national guidelines on PA in childhood have pointed to the beneficial effects of regular PA by children on the development of their emotional, cognitive, and physical health (51–57). However, understanding the determinants of PA in children has remained incomplete. Motor development is often cited as one of the most important determinants of sufficient PA in childhood (58,59). So far, the idea that motor development is a causal factor for future PA of the child is based on studies that demonstrate a prospective association between motor skill development and future PA (59). However, establishing such prospective associations does not rule out confounding by genetic or shared environmental factors that could independently influence motor skill development and PA. The substantial genetic and shared environmental effects found on motor milestones here, paired to extensive evidence that such factors also operate on childhood PA (30,60,61), demonstrate that such confounding is a real possibility. Bivariate twin models of motor development traits and childhood an adolescent PA could help resolve this question.

### Strengths and limitations

The main limitation is the potential for bias in the mother reported motor milestones. Whereas motor development is one of the more objective changes during infancy (62), and most mothers remember their child’s first step well, the ability to accurately recall the month in which a child meets other landmarks, such as sitting without support, may be harder (63). Reporting bias would be specifically detrimental to the results if it was moderated by zygosity. If mothers of MZ twins are more inclined to recall motor development of their twins as being more similar because MZ twins resemble each other more than DZ twins, for example, in the trajectory of weight and height growth, this would overestimate heritability. To minimize reporting bias, we used a prospective approach in which mothers were send a memory aid in the months after birth. A one-page notebook with a list of the motor milestones was given. Here they could note down the month as soon as the milestone was reached. By tracking the development of early motor skills at the moment they occurred, the retrospective bias was minimized for mothers who copied the milestones from this sheet when filling out the survey on the milestones when the child was around 2 yr old. We cannot exclude that some mothers nonetheless relied on memory recall. Furthermore, even when they did use note taking, the perception of what properly constitutes “first crawling” or “standing without support” may still suffer from biases in subjective interpretation. However, comparison of the data obtained by this prospective recording method against data obtained by monthly telephone interviews repeated from months 6 to 20 showed excellent agreement for four out of five milestones, with only standing without support being reported to occur slightly earlier in the surveys than by telephone interview (29).

Another potential limitation of this study is the use of twins, who related to their rapid catch-up trajectory to make up for the low birth weight, may show deviant motor development compared with nontwins. However, the results of a previous study showed that the motor milestone development in twins could be generalized to singleton populations (64). As a third limitation, we acknowledge that MZ twins are in part monochorionic (~60%) and in part dichorionic, which could affect their resemblance for motor milestones. We did not take chorionicity into account, but a previous study suggested no effect on the intraclass twin correlations for motor milestones, save a very small effect on standing alone (65). Another limitation is that we have no information on parental PA patterns at the time of infants’ motor milestones achievement. Parents pass on their genes (i.e., genetic ability to be higher physically active due to more advanced motor development) to their children, and the physically active parents might also be more likely to create a home environment supporting these genetic tendencies. We could not detect the contribution of this effect to the shared environment found in this study.

Apart from its prospective design, a major strength of this study is its large sample size, which allowed us to present sex-specific estimates with narrow confidence intervals, and also enables detection of (sex-specific) moderator effects, such as that of parental educational attainment. Furthermore, our findings are based on a compound score that used multiple motor milestones, which captured early motor development more comprehensively, while also reducing effects of reporting bias on single items.

## CONCLUSIONS

Genetics is a significant determinant of variation in the early-life motor developmental milestones, but a large role is also played by the home environment which includes the parents education attainment level and the infant’s physical living environment.
